# Applications of ambient ionization mass spectrometry in 2020: An annual review

**DOI:** 10.1002/ansa.202000135

**Published:** 2021-02-06

**Authors:** Stephanie Rankin‐Turner, Liam M. Heaney

**Affiliations:** ^1^ W. Harry Feinstone Department of Molecular Microbiology and Immunology, Johns Hopkins Bloomberg School of Public Health Johns Hopkins University Baltimore Maryland USA; ^2^ School of Sport, Exercise and Health Sciences Loughborough University Loughborough Leicestershire UK

**Keywords:** Ambient ionization, atmospheric pressure ionization, high throughput analysis, mass spectrometry

## Abstract

Recent developments in mass spectrometry (MS) analyses have seen a concerted effort to reduce the complexity of analytical workflows through the simplification (or removal) of sample preparation and the shortening of run‐to‐run analysis times. Ambient ionization mass spectrometry (AIMS) is an exemplar MS‐based technology that has swiftly developed into a popular and powerful tool in analytical science. This increase in interest and demonstrable applications is down to its capacity to enable the rapid analysis of a diverse range of samples, typically in their native state or following a minimalistic sample preparation approach. The field of AIMS is constantly improving and expanding, with developments of powerful and novel techniques, improvements to existing instrumentation, and exciting new applications added with each year that passes. This annual review provides an overview of applications of AIMS techniques over the past year (2020), with a particular focus on the application of AIMS in a number of key fields of research including biomedical sciences, forensics and security, food sciences, the environment, and chemical synthesis. Novel ambient ionization techniques are introduced, including picolitre pressure‐probe electrospray ionization and fiber spray ionization, in addition to modifications and improvements to existing techniques such as hand‐held devices for ease of use, and USB‐powered ion sources for on‐site analysis. In all, the information provided in this review supports the view that AIMS has become a leading approach in MS‐based analyses and that improvements to existing methods, alongside the development of novel approaches, will continue across the foreseeable future.

## 1 INTRODUCTION

Almost 20 years ago, a powerful new class of mass spectrometry was introduced which revolutionized the field of analytical chemistry. In 2003, Cooks *et al* presented desorption electrospray ionization (DESI), an ionization technique capable of achieving the combined desorption and ionization of analytes directly from the surface of an untreated sample.^1^ This was closely followed by direct analysis in real time (DART), a plasma‐based ambient ionization technique introduced by Laramee and Cody in 2004.^2^ Through these two radical new techniques, the exciting field of ambient ionization was born.

Ambient ionization mass spectrometry (AIMS) enables the rapid analysis of samples in their native state, typically requiring minimal or no sample preparation.^3^ In traditional mass spectrometry techniques, analytes are introduced into a vacuumed region prior to ionization. This is contrary to ambient techniques, in which ions are formed outside of the mass spectrometer under atmospheric conditions and subsequently drawn into the instrument for mass analysis. This makes AIMS particularly applicable to *in situ* analysis through the unique benefit of enabling direct analysis of intact substances. Since the introduction of DESI and DART the field of AIMS has exploded, with the subsequent development of dozens of additional techniques and application of these methods across a wide range of fields. This is evident through the addition of around fifty or more scientific papers published on the topic annually since 2013 (MEDLINE search 14 October 2020). Furthermore, the advent of miniaturized or portable mass spectrometers offers another interesting route of development, opening up the possibility of achieving near‐real‐time chemical profiling in remote locations away from the laboratory.^4^ This has piqued particular interest in the clinical and forensic fields, both of which would greatly benefit from the ability to perform rapid, on‐site analysis of patient samples and forensic evidence, respectively. Although such technology has a long road of development before achieving the capabilities of benchtop instrumentation, the combination of ambient ionization with portable analyzers opens up an exciting world of possibilities in rapid, on‐site analysis across a range of fields.

This review will provide an overview of studies utilizing AIMS across key fields of research published in 2020 or currently in press. This is not intended to be a systematic review, but an overview of common and novel applications. Search terms included ‘ambient ionization’ and individual technique names primarily using the PubMed database in September 2020. Technological developments will not be the focus of this review, though a brief overview of the techniques covered in this review will be included. It is worth noting that a different yet closely related class of techniques, atmospheric pressure ionization (API) techniques, do not technically fall under the umbrella of ambient ionization. API techniques do operate under ambient conditions, but require some form of sample treatment prior to direct analysis of the sample.^5^ Some techniques covered in this review, such as MALDI, are not ambient ionization techniques, but are still included to ensure a complete overview of direct analysis applications.

## 2 AMBIENT IONIZATION MASS SPECTROMETRY TECHNIQUES

AIMS techniques typically fall into three primary classes. Solid‐liquid extraction techniques involve the extraction or desorption of molecules from the surface of a sample, generally achieving ionization by means of an electrospray ionization (ESI) mechanism.^6^ Plasma desorption techniques achieve ionization using plasma, with mechanisms akin to those in atmospheric pressure chemical ionization (APCI).^7^ Finally, laser ablation techniques use infrared or ultraviolet lasers to ablate and desorb analytes from the sample surface.^8^ Each of these categories has expanded with the development of atmospheric and AIMS techniques, covering the analysis of a range of sample types from small volatile compounds to large intact biomolecules.

The first and perhaps most well‐known electrospray‐based AIMS technique is DESI, which utilizes a charged stream of microdroplets directed at the surface of a sample.^1^ As the solvent stream collides with the surface, secondary droplets carrying desorbed and ionized analytes are propelled from the sample and towards the mass spectrometer. This results in mass spectra that generally resemble those produced by ESI, typically exhibiting singly or multiply charged molecular ions. Nanospray desorption electrospray ionization (nanoDESI) is a smaller‐scale ESI‐based technique that allows for highly localized surface sampling.^9^ nanoDESI uses two capillaries that form a liquid micro‐junction at the surface of the sample and, unlike DESI, generates charged droplets without the use of a nebulizing gas. Easy ambient sonic‐spray ionization (EASI) shares a similar setup to DESI, but without using heat or high voltages.^10^ In EASI, analyte ionization is based on a sonic spray, creating charged droplets using only a spray solvent and compressed nitrogen. EASI is not as widely utilized as techniques such as DESI, although it is considered a more simple method than other similar AIMS techniques.

Probe electrospray ionization (PESI) is an ESI‐based technique initially developed by Hiraoka et al.^11^ PESI utilizes a grounded solid needle, typically composed of stainless steel or titanium, which is briefly touched to the surface of a sample, transferring a small amount of sample to the needle tip. The needle is positioned in front of the mass spectrometer, either manually or robotically, and a high voltage is applied to induce electrospray from the tip of the needle. A modification of this technique, sheath‐flow PESI (sfPESI) enables the application of PESI to solid materials by enclosing the needle in a solvent‐filled sheath to enable surface analyte extraction.^12^ This technique is somewhat comparable to liquid extraction surface analysis (LESA), developed by van Berkel *et al*, which also achieves the surface extraction and ionization of analytes directly from a material.^13^ In LESA, a small volume of solvent is robotically applied to the sample surface, and a repeated dispense/extract cycle occurs, enabling the extraction of analytes into the solvent. Upon the final aspiration of the solvent, the extracted analytes are electrosprayed toward the mass spectrometer inlet using the Advion NanoMate nano ESI system. Previous research has demonstrated that sfPESI and LESA can achieve similar results,^14^ though sfPESI may be the more cost‐efficient approach, not requiring the purchase of additional instrumentation such as the NanoMate system.

Paper spray ionization (PSI) uses a triangular‐shaped piece of paper onto which the sample is applied.^15^ After sample drying, a spray solvent is added to the substrate and a high voltage is applied to the paper, inducing electrospray of the sample from the tip of the triangle. Similar to the aforementioned techniques, PSI is based on ESI mechanisms, though with an important difference. Rather than being a solid‐liquid extraction method, PSI generates ions directly from the paper substrate onto which the sample has been deposited. Other materials can be used as the substrate, such as plant material as in leaf spray^16^ and polymers,^17^ however paper is most commonly employed. Extractive electrospray ionization (EESI) utilizes two individual sprayers; one to spray the solvent and modifiers and the other to nebulize the sample solution.^18^ A high voltage is applied to the solvent sprayer, producing charged microdroplets, whilst the sample sprayer remains grounded but utilizes a stream of nitrogen to aid sample nebulization. The two sprayers are positioned in front of the mass spectrometer inlet in such a way that the nozzles intersect, enabling ionization as charge from the solvent molecules is transferred to the analytes. Somewhat similar to EESI is SESI, or secondary electrospray ionization. This technique also achieves analyte extraction and ionization using an electrospray plume, but incorporates gas phase samples as opposed to nebulized liquid samples.^19^

A popular plasma‐based AIMS technique is DART, which was developed around the same time as DESI but achieves direct desorption and ionization of analytes using a plasma‐based ion source.^2^ In DART, an electrical potential is applied to a flow of helium or nitrogen gas. This induces the formation of a plasma containing various metastable species, ions, and electrons, which results in the desorption of analytes directly from the sample surface. In the DART source, various ionization processes occur, including proton transfer when helium is used as the gas and Penning ionization when nitrogen or neon are used.^20^

The atmospheric pressure solids analysis probe, or ASAP, utilizes a sampling probe that collects and exposes the sample directly to the plasma of a modified APCI source.^21^ Analyte desorption occurs by a stream of heated gas, and analyte ionization mechanisms are similar to those taking place in a typical APCI ion source. The simple design and ease of use of ASAP make it an ideal candidate for combination with portable mass spectrometers for rapid on‐site analysis, though as ASAP was developed for the analysis of liquids and powders, it is not readily applied to surface analysis. Some plasma‐based techniques specifically utilize a low‐temperature plasma. Dielectric barrier discharge (DBD) involves the application of a high‐voltage current between two electrodes, such as a needle and a copper strip, separated by an insulating barrier, typically glass.^22^ This results in the formation of a low‐temperature plasma to which a sample can be directly exposed to induce desorption and ionization. Low‐temperature plasma (LTP) is a similar DBD‐based technique, but uses a different placement of counter electrodes that make the technique more amenable to direct, on‐site analysis.^23^

Laser ablation techniques offer the primary benefit of achieving highly focused sampling for the reduction of spatial resolution.^8^ Laser ablation electrospray ionization (LAESI) uses an infrared laser to ablate the surface of a sample and produce a plume of desorbed molecules.^8^ Analytes contained within this plume are subsequently ionized by ESI and ions are drawn into the mass spectrometer for analysis. As a similar alternative, laser ablation atmospheric pressure photoionization (LAAPPI) also utilizes an infrared laser to ablate the sample surface, but incorporates an atmospheric pressure photoionization source for ionization of desorbed analytes.^24^ Matrix‐assisted laser desorption/ionization (MALDI), although an atmospheric pressure ionization technique as opposed to an ambient method, has rapidly become a popular technique in direct analysis. In MALDI, the sample is mixed with a matrix and the mixture is applied to a plate. A pulsed laser is then used to ablate the sample and desorb analytes, which are subsequently ionized and drawn into the mass spectrometer for analysis.^25^ This soft ionization technique has proven crucial in the analysis of large intact biological molecules such as proteins and peptides. Following the introduction of MALDI, an atmospheric pressure alternative (AP‐MALDI) was subsequently developed, which enables analysis by MALDI‐MS without the need for placing the sample in a vacuum.^25^ As a similar technique, matrix‐assisted laser desorption electrospray ionization (MALDESI) was developed to combine aspects of MALDI with an ESI source.^26^ Samples are crystallized in an organic matrix in preparation for laser ablation, after which ionization of the sample plume occurs using an ESI source. More recent developments have utilized a thin ice layer as the matrix and a mid‐infrared laser for ablation.^27^

As the field of ambient ionization expands, further ionization sources have been developed that fall outside of the three primary categories previously described, such as those utilizing different ionization mechanisms or integrating different systems. Rapid evaporative ionization mass spectrometry (REIMS) was initially developed to guide surgeons via the real‐time characterization of human tissue during medical procedures, specifically the differentiation between healthy and cancerous tissue.^28^ During surgery, an electrocautery knife is used for the vaporization and subsequent ionization of human body tissue. The ionized analytes are drawn into the mass spectrometer for analysis, after which mass spectra are compared to a database of healthy and cancerous reference spectra for the near‐real‐time assessment of tissue health.

**FIGURE 1:**
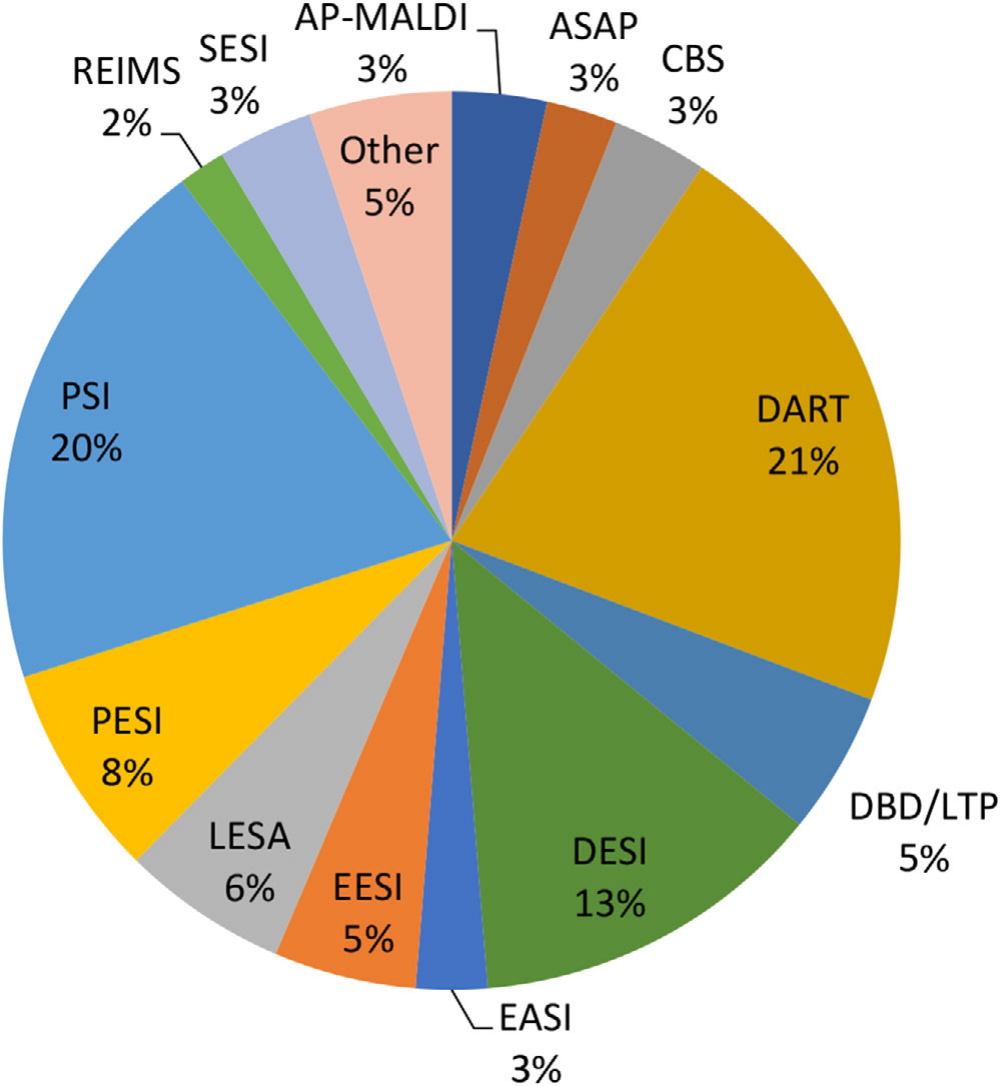
Percentage of papers covered in this annual review utilizing each AIMS technique

The AIMS techniques discussed here and the percentage of 2020 publications using each technique are summarized in Table 1 and Figure 1, respectively. As a result of the rapid and cost‐effective nature of ambient ionization methods and the broad range of versatile techniques available, AIMS has been successfully demonstrated in a diverse range of research areas. The following sections will discuss recent applications of AIMS in a number of key fields of research.

**TABLE 1:** Summary of techniques covered in this annual review

Technique	Abbreviation	Classification
Atmospheric pressure matrix‐assisted laser desorption/ionization	AP‐MALDI	Ablation
Atmospheric solids analysis probe	ASAP	Thermal desorption
Coated blade spray	CBS	Liquid extraction
Desorption electrospray ionization	DESI	Liquid extraction
Dielectric barrier discharge	DBD	Plasma desorption
Direct analysis in real time	DART	Plasma desorption
Easy ambient sonic‐spray ionization	EASI	Liquid extraction
Extractive electrospray ionization	EESI	Liquid extraction
Fiber spray ionization	FSI	Liquid extraction
Laser ablation atmospheric pressure photoionization	LAAPPI	Ablation
Laser ablation electrospray ionization	LAESI	Ablation
Liquid extraction surface analysis	LESA	Liquid extraction
Low‐temperature plasma	LTP	Plasma desorption
Matrix‐assisted laser desorption electrospray ionization	MALDESI	Ablation
Nano‐desorption electrospray ionization	nano‐DESI	Electrospray
Packed ballpoint‐electrospray ionization	PBP‐ESI	Liquid extraction
Paper spray ionization	PSI	Liquid extraction
Probe electrospray ionization	PESI	Liquid extraction
Rapid evaporative ionization mass spectrometry	REIMS	Thermal desorption
Secondary electrospray ionization	SESI	Liquid extraction
Thread spray ionization	TSI	Liquid extraction

## 3 APPLICATIONS OF AMBIENT IONIZATION MASS SPECTROMETRY

### 3.1 Biomedical applications: Disease diagnostics

Clinical analysis is undoubtedly one of the largest fields of application for AIMS, both within disease diagnostics and therapeutic drug monitoring. The high‐throughput nature of AIMS, along with its compatibility with complex matrices, has logically resulted in the widespread use of such techniques for the identification of potential disease biomarkers and the development of protocols for disease diagnostics, with the aim of supplementing or replacing time‐consuming clinical methods currently in use.

PESI‐MS has great potential for the rapid analysis of biological tissues in clinical analysis. Giordano *et al* applied PESI‐MS to the analysis of over 300 tumor carcinoma samples, consisting of both hepatocellular carcinoma and angiocarcinoma.^29^ Artificial intelligence algorithms were developed for the differentiation between acquired mass spectra from tumor and non‐tumor tissue, and between different types of tumor tissue. Overall, diagnostic accuracy was greater than 94%. Furthermore, PESI‐MS has been utilized for the rapid lipid profiling of breast cancer tissue, again to differentiate between healthy and cancerous tissue.^30^ Partial least squares discriminant analysis (PLS‐DA) enabled the differentiation between the two sample groups and the identification of a number of lipids, namely phosphatidylcholines (PCs) and phosphatidylethanolamines, that could be candidate breast cancer biomarkers. A similar PESI‐MS and multivariate analysis approach was also applied to the analysis of serum from patients with pancreatic cancer and healthy subjects with a family history of pancreatic cancer, analyzing almost 600 serum samples in total.^31^ The technique exhibited a diagnostic accuracy of over 91% and not only enabled the differentiation between healthy controls and cancer patients, but also differentiated between different stages of pancreatic cancer. This demonstrates the potential of PESI‐MS to not only diagnose developed disease, but also potentially improve early disease detection and prognostication.

Conductive polymer spray ionization mass spectrometry (CPSI‐MS), a modification of paper spray ionization, was utilized for the detection of biomarkers indicative of oral squamous cell carcinoma.^32^ Saliva samples were collected from a cohort of 373 individuals and all analyzed within 4.5 h, resulting in the detection of over 600 common peaks across samples. A machine learning model was used for the study and prediction of carcinoma status based on mass spectral profiles. Slight metabolic changes were detected in cancer‐positive samples, including changes related to numerous pathways, such as tRNA biosynthesis, histidine and proline metabolism, and arginine biosynthesis. Based on these metabolic differences, the method was able to differentiate malignant and premalignant samples from healthy samples with an accuracy of 86.7%. PSI has also been applied in the diagnosis of cervical cancer.^33^ A total of 86 plasma samples from patients with cervical cancer and healthy controls were collected, and 10 μL of untreated plasma was analyzed by PSI‐MS. Linear discriminant analysis was applied to acquired spectra, and six ions of interest were identified that enabled differentiation between diseased and healthy patients. Numerous other studies have also investigated the potential of paper spray ionization MS as a tool for rapid cancer diagnostics. Huang *et al* coupled paper spray with ion mobility spectrometry for the analysis of breast cancer tissue,^34^ and Chen *et al* coupled PSI‐MS with a lab‐on‐membrane platform for the analysis of prostate‐specific antigen, a glycoprotein implicated in prostate cancer and commonly used as a biomarker for clinical screening.^35^ As a slightly different approach, a microfluidic paper‐based analytical device (μPAD) was coupled with PSI‐MS for the detection of C_18_‐ceramide in serum.^36^ Ceramides are lipids present in the cell membranes of eukaryotic cells, and have been associated with several diseases, including cancer and Alzheimer's disease.

Mass spectrometry imaging (MSI) has become particularly important in the study of potentially diseased tissues and organs. Vijayalakshmi *et al* applied DESI‐MSI to the analysis of clear cell renal cell carcinoma (ccRCC) tissue, detecting lipids, fatty acids, and other small metabolites.^37^ From a suite of over 27 000 metabolic features, 57 peaks were detected via Lasso analysis. Tissue samples could be classified as either normal or cancerous using a predictive model, with an accuracy of 94% and 76%, respectively. Robison *et al* developed a DESI‐MS method for the identification of lipids related to breast cancer.^38^ Human epidermal growth factor 2 (HER2) is correlated with the mutation of tumor suppressor p53 and increased metastatic potential. The study examined cancerous and non‐cancerous cell cultures to identify lipids indicative of HER2 expression, and then went on to use DESI imaging to map the spatial distribution of lipids of interest in metastatic spheroids, identifying three lipids of interest.

Triglycerides (TGs) are a class of lipid abundant in many biological systems, and abnormal lipid levels in human plasma have been associated with a range of diseases, including coronary heart disease, liver disease, and type‐2 diabetes.^39^ Given the obvious importance of this class of metabolites, the development of analytical techniques for the fast and reliable analysis of such molecules has important implications in disease diagnostics. Although lipids are of great interest, the analysis of lipids such as TGs can be challenging due to ion suppression effects primarily caused by the presence of PCs.^39^ Unsihuay *et al* took a nano‐DESI approach to the imaging of TGs in murine tissues, with an aim to establish the optimum conditions for TG detection whilst also reducing problems of ion suppression.^40^ By exploring different solvent compositions and ionic dopants, the study established that TGs are primarily detected as potassium adducts and are most readily extracted using less polar solvent mixtures.

Since the COVID‐19 pandemic, the need for rapid and high‐throughput diagnostic testing strategies has become more apparent than ever before. In a recent proof‐of‐concept study, paper spray ionization was utilized for the rapid analysis of COVID‐19 positive and negative swab samples, as confirmed by PCR.^41^ Teslin®, a microporous polyolefin‐silica matrix, was used as the paper substrate to improve analyte sensitivity. Numerous metabolites were altered in samples from COVID‐19 positive patients. Seventeen lipids were significantly upregulated in positive samples, including PCs, phosphatidylserines, and diglycerides. Downregulated metabolites included dihydrouracil, ʟ‐malic acid, glycerol‐3‐phosphate, and guanosine monophosphate. Mass spectra were processed using linear discriminant analysis, showing detectable discrimination between positive and negative sample groups. Based on this statistical analysis, there was a 93.3% correlation between the analysis by PSI‐MS and the traditional PCR method.

As analytical techniques for rapid disease diagnostics develop, the need for non‐invasive sampling protocols is imperative. Exhaled breath is a prime target as a non‐invasive sample medium, and is known to contain hundreds of compounds, some of which have been implicated in various diseases.^42^ Wu *et al* utilized EESI‐MS in the analysis of exhaled breath for the characterization of liver failure.^43^ Twenty liver failure patients, 20 chronic hepatitis B patients, and 24 healthy controls provided exhaled breath samples for the study, and principal component analysis (PCA) was applied to study the chemical differences between the sample groups. Although healthy controls and chronic hepatitis patients were not readily differentiated, samples from liver failure patients formed a tight cluster in the PCA plot, indicating potentially significant metabolic differences in the breath of these patients. Twenty‐two ions were found to differ significantly between the liver failure and healthy control groups, eight of which were identified, including 1‐butylene, butanone, 2‐ethylacrylic acid, and 2,3‐octanedione. Similarly, SESI‐MS is a commonly reported technique in breath analysis. Two recent studies have applied SESI to the real‐time profiling of breath metabolites following the ingestion of peppermint oil, which is commonly used in the field of breath analysis for benchmarking the performance of different instruments.^44^, ^45^ Both studies detected a broad range of peppermint‐related metabolites, particularly monoterpenes. Although targeting the presence of exogenous compounds, the studies demonstrate the potential strength of SESI‐MS for rapid, non‐invasive metabolomics.

Recently, the use of face masks for protection against bacteria, viruses, and other environmental contaminants has become more commonplace. This offers a novel potential route of breath sampling. Yuan *et al* inserted SPME fibers into face masks for the collection of exhaled breath aerosol, followed by direct analysis using DART‐MS.^46^ Numerous ions related to diet and lifestyle habits were detected, such as protonated nicotine and cotinine derived from tobacco use at *m/z* 163 and 177, respectively. Protonated caffeine was detected at *m/z* 195 in the breath of a participant shortly following coffee consumption, and salbutamol and metronidazole were detected following the use of an asthma aerosol drug. Although this study primarily focused on the detection of exogenous metabolites, this simple and non‐invasive approach has the potential to be utilized in the detection and monitoring of disease biomarkers.

### 3.2 Biomedical applications: Drug monitoring

Therapeutic drug monitoring is required for the study of administered medicines to ensure optimal levels of delivery and efficacy. This allows for the monitoring of circulating drug (or metabolite) levels to ensure patient adherence to medications, and to assess cases of possible medication overdose. Traditional analytical techniques can be time‐consuming, thus not ideal in situations in which rapid and real‐time drug monitoring is imperative.

Anti‐arrhythmic drugs are prescribed to patients suffering from an irregular heart rhythm, however common medications in this category can have a very narrow therapeutic window that can differ between individuals. For this reason, the monitoring of anti‐arrhythmic drugs and their metabolites in the body is essential to ensure the appropriate administration. DART‐MS/MS was recently applied to the rapid determination of several anti‐arrhythmic drugs in serum, including amiodarone, propafenone, and verapamil.^47^ High‐throughput serum analysis of approximately 30 seconds per sample was achieved and, with the use of a stable isotope‐labeled internal standard, arrhythmic drugs and metabolites could be detected and quantified with good precision, accuracy and linearity. Similarly, the prostate cancer drug abiraterone requires optimization to individual patients to ensure the administration of appropriate dosages. Bhatnagar *et al* applied PSI coupled with tandem MS to the detection and quantification of abiraterone in patient plasma.^48^ The technique showed good repeatability, with the RSD typically below 10%. It also exhibited a similar performance to that achieved by more traditional liquid chromatography (LC)‐MS/MS approaches, demonstrating PSI‐MS to be a suitable potential alternative to the slower and more costly analytical techniques. PSI is rapidly becoming the ambient technique of choice for therapeutic drug monitoring, additionally proving effective in the quantification of triazole antifungal agents in plasma,^49^ and for the detection of tricyclic antidepressants in urine.^50^ CBS‐MS has also been utilized in therapeutic drug monitoring for the detection of immunosuppressive drugs in whole blood samples.^51^ Samples were measured by CBS coupled with a triple quadrupole mass spectrometer, achieving limits of detection at the ng/mL level for various types of immunosuppressant, though sample preparation was required prior to analysis.

Fu *et al* proposed a novel technique for the analysis of complex biofluids. Packed ballpoint‐electrospray ionization (PBP‐ESI) incorporates a hollow ballpoint pen chamber packed with C18 adsorbent material.^52^ A spray solvent is pumped through the device, and biofluid passed through the adsorbent. A wire is wrapped around the outer surface of the device, inducing electrospray at the tip. In this recent study, blood and urine spiked with various therapeutic drugs, including aripiprazole, verapamil, and citalopram, were analyzed. The study described an interesting new approach to rapid biofluid analysis for therapeutic monitoring, with the limit of quantification for all drugs being between 0.3 and 1 ng/mL, and precision between 1.9% and 16.1%.

A somewhat different application of AIMS in drug monitoring is in sports science to support anti‐doping efforts. In professional competitive sports, the World Anti‐Doping Agency (WADA) maintains an extensive list of performance‐enhancing drugs that professional athletes are forbidden from using, and there is a great need for the development of rapid and accurate analytical techniques to keep up with demand in monitoring drug use among athletes. PSI has great potential for this application. Gorgens *et al* recently demonstrated the use of PSI‐MS for the detection of prohibited compounds in blood and urine, with a particular focus on polar compounds.^53^ In this study, raw blood and urine were spotted onto the paper substrate and subjected to direct analysis. A number of “model compounds” were investigated in this proof‐of‐concept study, including meldonium, metformin, bemitil, and tramadol, all drugs of interest in competitive sports. The protocol enabled the rapid detection of all compounds of interest, with all but one of the drugs meeting WADA's minimum required performance limits for analytical testing protocols. The study furthermore combined the analysis with field asymmetric ion mobility spectrometry (FAIMS) to further improve assay sensitivity and selectivity. Rossini *et al* took a similar approach, applying hydrophobic PSI to the detection of doping agents in raw urine.^54^ In this study, the paper surface was treated with trichloromethylsilane to improve the ionization of target analytes, specifically the anabolic agents clenbuterol and trenbolone, and the diuretics furosemide and hydrochlorothiazide. Following optimization, detection limits in the sub‐nanogram per milliliter levels were achieved, below WADA's minimum required limits of detection (LODs).

### 3.3 Forensics and security

The field of forensic science has benefited greatly from the development of AIMS techniques. Preservation of materials pertinent to criminal investigations and national security is of the utmost importance, particularly should further analysis be required. Traditional analytical techniques commonly utilized in forensic science, such as gas chromatography (GC)‐ and LC‐MS, require invasive sample preparation and ultimately result in the destruction of the sample. This can be particularly problematic if only trace amounts of material are available for analysis. AIMS techniques are often non‐ or minimally destructive to the sample, thus have been widely explored for use in the analysis of forensic evidence.

Perhaps the most common application of ambient ionization in forensic science is that of rapid drug detection. Identification of suspected illicit substances typically involves the use of chemical presumptive tests and transport to a laboratory for analysis by GC‐MS. A range of AIMS techniques have been applied to the rapid identification of controlled or adulterated drugs, including DART,^55^, ^56^, ^57^, ^58^ PSI and its variants,^59^, ^60^, ^61^, ^62^, ^63^, ^64^ ASAP,^65^ and REIMS.^66^ The coupling of ambient ionization sources with portable mass analyzers demonstrates the potential for rapid, on‐site analysis of suspected illicit substances. McCullough *et al* coupled ASAP with a miniaturized single quadrupole mass spectrometer for the analysis of 50 drugs of abuse common to the UK, including cocaine, cannabis, MDMA, and ketamine, showing drug identification with an analysis time of as little as 2 min.^67^ The simplicity of ASAP and its compatibility with portable instrumentation would make this a prime candidate for use by police forces for rapid on‐site analysis.

**FIGURE 2:**
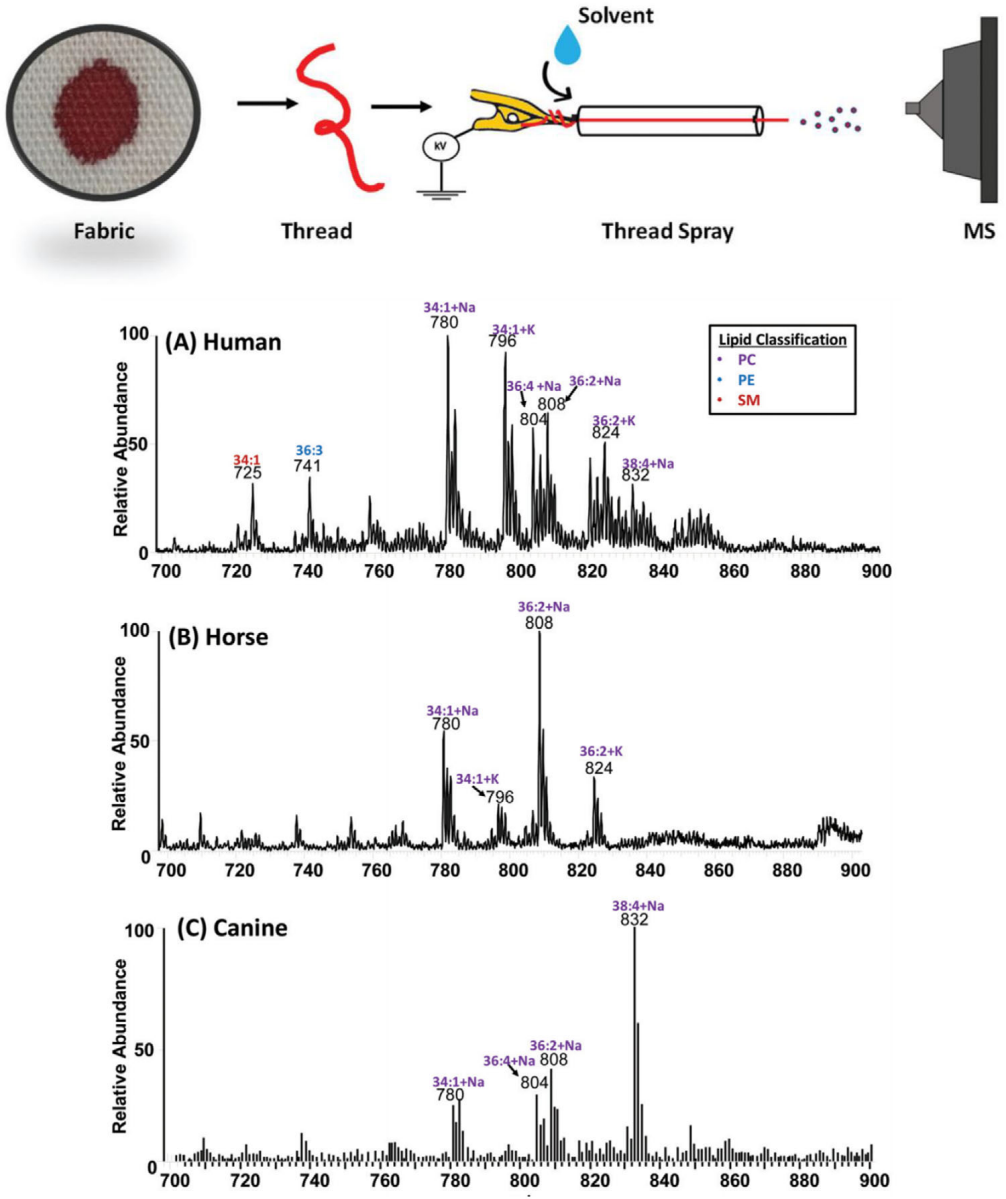
The experimental setup of thread spray mass spectrometry and the lipid profiles from human (A), horse (B), and canine (C) blood. Reproduced with permission from The Royal Society of Chemistry from Jackson et al 2020^68^

The forensic analysis of body fluids is applied for both body fluid identification and for the detection of illicit substances to demonstrate drug use. Jackson *et al* applied thread spray mass spectrometry to the analysis of bloodstained textiles. Thread spray is a recently developed technique in which a high voltage is applied to a single fabric thread, inducing electrospray directly from the tip of the thread (Figure 2). Numerous large biological molecules could be detected that were indicative of blood, including hemoglobin, demonstrating the possibility of performing rapid analyses of stained fabrics to confirm the presence of blood.^68^ Furthermore, variations in lipid profiles were detected in human, horse, and canine blood, indicating the potential to determine whether or not a bloodstain is of human origin (Figure 2). Although an interesting technique, this method is only pertinent to the analysis of samples applied to fabric materials, severely limiting its practical applications. Probe electrospray ionization and associated techniques have been utilized for both body fluid identification and for the detection of exogenous compounds in biofluids. Sheath‐flow‐PESI was recently applied to the analysis of sexual assault evidence, demonstrating the first use of AIMS in the analysis of semen, and the ability to confirm the presence of both fresh and aged semen on a range of surface materials.^69^ The study also applied the technique to the analysis of condoms, including the applied lubricants, flavorings, and spermicides, indicating the wealth of potentially useful chemical evidence that can be obtained from prophylactics.

**FIGURE 3:**
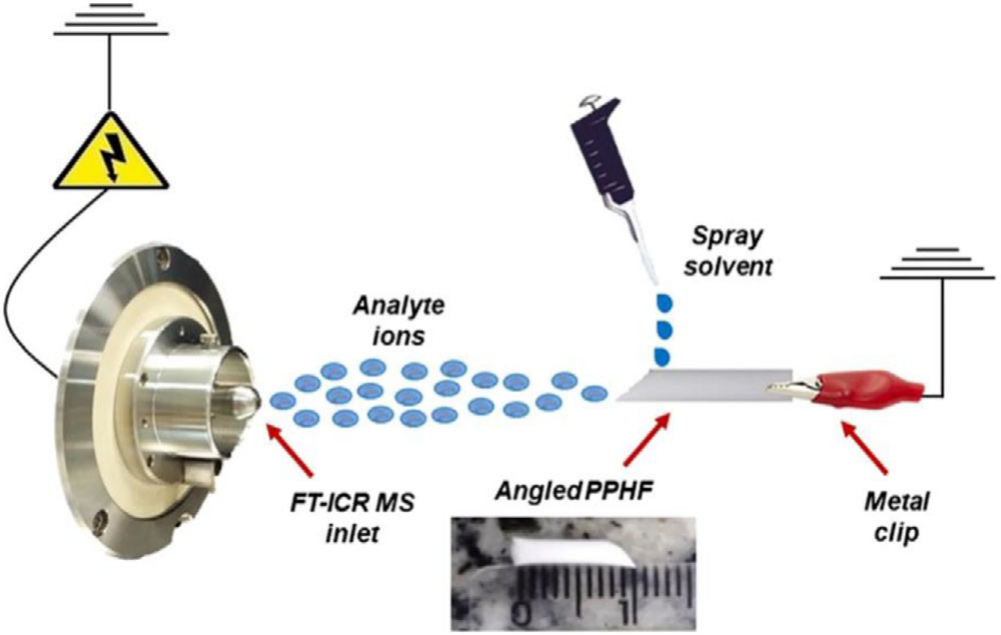
A schematic representation of fiber spray ionization mass spectrometry. Reproduced with permission from John Wiley and Sons from Filho *et al* 2020^72^

Hisastune *et al* applied PESI‐MS to the analysis of human whole blood for the purpose of detecting cyanide and 2‐aminothiazoline‐4‐carboxylic acid, compounds indicative of cyanide poisoning. The technique achieved detection limits of 42 and 43 ng/mL, respectively.^70^ DART‐MS has been applied to the detection of exogenous materials in body fluids. Liang *et al* used transmission mode‐DART‐MS, in which microliter samples of biofluid are first deposited onto a wire mesh substrate prior to exposure to the DART ion source, enabling reproducible sample positioning.^71^ The technique was able to successfully detect both codeine and methadone in raw urine. Fiber spray ionization (FSI)‐MS has also been applied to the detection of cocaine in urine.^72^ FSI utilized a capillary polypropylene hollow fiber, which was dipped directly into the lipid sample. Following sampling, the fiber was positioned in front of the MS inlet and a high voltage applied to induce ionization (Figure 3). The technique was able to detect cocaine in urine down to an LOD of 5.16 ng/mL and with repeatability below 3% RSD, in addition to metabolites such as benzoylecgonine and cocaethylene.

A particularly interesting application of DART is in the field of forensic entomology to identify forensically important insects. During the investigation of a suspicious death, the presence and age of specific insects can provide insight into the post‐mortem interval, or time since death. DART coupled with high‐resolution mass spectrometry (HRMS) was recently used for rapid species determination of maggots of various fly species.^73^ By applying machine learning to mass spectral profiles, mixtures of up to six different fly species could be identified with confidence limits up to 99%. This is of particular interest given that mixed species are often encountered at the scene of a death, and there are currently no rapid methods for species identification.

Finally, in recent years analytical technologies have been applied to the analysis of products susceptible to counterfeiting. Agarwood is a valuable resinous wood widely utilized in Chinese culture, particularly in perfumes, medicines, and decorations. Xie *et al* proposed the use of LESA‐MS for the rapid authentication of agarwood.^74^ A total of 62 wood samples were subjected to analysis, followed by multivariate analysis to study characteristic differences between authentic and counterfeit materials. Fake and authentic agarwood were readily separated by PCA, and a number of characteristic ions were identified. Of particular importance was a compound detected at m/z 319, identified as 2‐(2‐phenylethyl)chromone, known to be present in agarwood. Although a niche application of AIMS for authentication purposes, the study highlights the potential of direct analysis mass spectrometry for the rapid detection of counterfeit goods. Other areas of forensic science have also seen applications of direct MS.

Low‐temperature plasma has been utilized for the direct detection of chemical warfare agents in soil,^75^ and DART‐MS for the quantification of ethanol in beverages and the rapid screening of explosives.^76^, ^77^ Although DART has been a popular ionization technique for explosives analysis, Mullen *et al* recently combined SESI‐MS with corona discharge ionization and drift tube ion mobility spectrometry.^78^ This combination of approaches achieved greater sensitivity for the detection of TNT and 2,6‐DNT vapors. DESI and EASI have both been applied to the analysis of adulterated documents, demonstrating that chemical differences in pen inks can be rapidly determined to aid in document authentication,^79^, ^80^ which also offers an exciting approach for the potential to identify counterfeit or adulterated artwork.

### 3.4 Food and agriculture

The food and agriculture industry requires rapid and high‐throughput testing strategies to enable the fast detection of food adulteration and contamination in order to ensure safety for human consumption. The nature of AIMS makes this suite of techniques particularly suited to this field of work, as demonstrated by the increasing use of ambient techniques within the field.

DART‐MS has unsurprisingly been widely applied in rapid food analysis due to its simple and user‐friendly interface, both for food safety monitoring and food metabolomics. Meng *at al* utilized DART coupled with an Orbitrap mass spectrometer to measure pyrethroids in food contact materials.^81^ Pyrethroids, such as permethrin and cyfluthrin, are a class of toxic synthetic compounds used for the preservation of wood, which may subsequently be used as food packaging and utensils, raising significant health concerns. Using this technique with a deuterated isotopic internal standard for quantification, limits of detection down to 0.04 mg/kg and relative standard deviations as low as 5.2% were achieved, though some sample preparation was required for enrichment and purification prior to analysis. In a similar study, DART was used in the rapid analysis of bamboo‐based biopolymers, which have recently become a popular choice of biodegradable food packaging materials.^82^ Dishes, jugs, and cups composed of the bamboo‐based polymer were analyzed by DART, SPME‐GC/MS, and UPLC‐QTOF‐MS. Amongst the compounds detected, numerous melamine derivatives were identified. Melamine is a non‐biodegradable additive commonly added to such packaging materials, and many of these derivatives were found at levels above limits determined by European legislation.

Mass spectrometry is widely utilized in the study of food and drink products for the assessment of flavor components, to study changes over time, and to generally investigate chemical differences between different product types. PSI has been used to determine the pungency of peppers, a characteristic that can be caused by capsaicinoids.^83^ Pepper slices from twelve types of pepper were either homogenized and solvent extracted prior to application to the paper substrate for analysis, or physically compressed against the paper to transfer compounds from the pepper. Capsaicin and dihydrocapsaicin were measured and ion intensities found to correlate with pepper pungency values, established using the Scoville scale. An inverse relationship was found between sugar content and pungency, with low sugar ion intensities observed in particularly pungent samples.

A recent study utilized DART coupled with TOF‐MS for the rapid differentiation of different strains of dried yeast.^84^ Yeast strains were suspended in a water/methanol solution and introduced to the DART ion source using melting point capillaries. Both positive and negative ion mode mass spectra were collected, and multivariate analysis used to differentiate the yeast types. Twelve strains of *Saccharomyces cerevisiae* and 5 strains of *Saccharomyces pastorianus* were differentiated, with certain metabolic differences noted depending on the type of yeast. Interestingly, discriminant analysis of principal components demonstrated clear separation between yeasts typically used for baking and the production of ales, lagers, and wines. DART‐MS has also been employed to study dried fruits used in traditional Chinese herbal medicine,^85^ for the authentication of fruit juices,^86^ and for the measurement of pharmaceutical drugs in meat products.^87^

Pesticides are toxic chemicals commonly applied to fruits and vegetables to protect the produce from insects, bacteria, and rodents. Given the link between pesticide exposure and numerous health disorders, there is a need to rapidly monitor pesticide levels in products intended for human consumption. In a study by Moura *et al*, acephate, cyazofamid, and chlorpyrifos, three insecticides commonly used in Brazil, were measured in tomato peels by PSI‐MS.^88^ Although this application required the homogenization and solvent extraction of samples prior to analysis, the authors demonstrated a rapid analysis technique with relative standard deviations below 9% and LODs of 0.01 ppm. Automated coated blade spray (CBS) tandem MS has also been applied to pesticide analysis, specifically in the detection of pesticides in various fruits, including blueberries, apples, and strawberries.^89^ Fruit matrices were cryoground to form a fine powder, diluted, and subjected to analysis by both CBS‐MS/MS and LC‐MS/MS for comparison of methods. A range of pesticides was detected at the nanogram per gram level in each type of fruit, with limits of quantification below the minimum regulatory limits achieved. CBS‐MS/MS significantly reduced the required sample amount and analysis time, and analysis of real‐world fruits by both CBS‐MS and LC‐MS yielded comparable results.

DESI‐MS has been applied to the study of amino acids in food, which are of particular interest in food chemistry due to the potential implications for both nutrition and safety.^90^ A repeatability‐enhancing modification of DESI, known as microfluidic voltage‐assisted liquid DESI, was applied to the analysis of *Cordyceps sinensis* and *militaris*, popular edible fungi available from local grocery stores. Numerous amino acids were simultaneously detected, including glutamine, arginine, tyrosine, tryptophan, and phenylalanine. The quantities detected were in line with literature values, suggesting the technique could be a suitable replacement for traditional analytical techniques.

There is also significant potential for ambient ionization mass spectrometry in the detection of food fraud and food authentication. The dairy industry has been the target of food fraud, particularly with the adulteration of milk from one animal with that of another. Typically, GC‐MS is used to confirm the origin of milk‐based on the presence of key lipids, however, this requires extensive sample preparation before the samples are amenable to analysis. Piras *et al* applied AP‐MALDI to the profiling of lipids and proteins of sheep and goat milk.^91^ The mass spectra for sheep and goat milk exhibited distinct chemical differences and, following linear discriminant analysis, a classification accuracy up to 100% could be achieved.

Antioxidants are substances that have been linked to the reduction of various diseases and health problems, and thus are occasionally added to food products to increase the health benefits related to consumption. However, if manufacturers are claiming the presence of specific concentrations of antioxidants in their products, robust analytical methods are required to assess the validity of such claims. PSI‐MS was recently utilized in the measurement of tyrosol and hydroxytyrosol in olive oil, two antioxidants often reported to offer health benefits to consumers.^92^ The authors demonstrated an accurate and sensitive technique for the rapid assessment of antioxidants in oil products, with the aim of upholding European regulations on food product health claims. REIMS, or the intelligent knife (iKnife), has primarily been utilized in the analysis of human tissue for clinical purposes, though in recent years new applications have emerged. Song *et al* applied REIMS to the analysis of untreated fish from four popular species of tuna, with the aim of developing a rapid technique for the authentication of tuna products.^93^ The mass spectra were dominated by a series of fatty acids and phospholipids which, when subjected to multivariate analysis, enabled the clear differentiation between the four species. Current methods for tracing the origin of honey involve a combination of uncertain techniques such as the study of pollen, and time‐consuming analytical techniques such as chromatography coupled with mass spectrometry. Gao *et al* applied EESI‐MS to the chemical analysis of honey and nectar products, with HPLC‐UV used to validate the results.^94^ EESI was demonstrated to be a reliable and rapid technique capable of detecting analytes in the complex matrices. Based on the presence of polyphenols and amino acids, honey products, and their nectar counterparts could be readily distinguished by PCA, though different types of honey could not be easily differentiated. These advancements provide the potential for the analytical identification for the origin and authentication of food products.

### 3.5 Environmental

Mass spectrometry has been an essential analytical tool in environmental sciences, enabling the identification and quantification of pollutants and contaminants in matrices such as soil, water, and vegetation. However, the analysis of environmental samples, such as water, may require the transportation of large sample volumes to the laboratory, followed by the extensive and time‐consuming clean‐up of the complex samples prior to analysis. The ability to perform on‐site analysis of raw samples could vastly improve environmental monitoring efforts, reducing both the need to transport samples to the laboratory and sample preparation time, as well as increasing the regularity of sampling and analysis protocols.

A common application of AIMS in environmental science is the detection of potentially harmful contaminants in water supplies. Jjunju *et al* utilized PSI‐MS for the direct detection of non‐conjugated steroids in water, specifically for the purpose of understanding potential hazards to marine populations.^95^ By applying raw aquaculture water samples to paper substrates for direct analysis, sub‐nanogram levels of aldosterone, corticosterone, cortisol, and β‐estrone were detected. Similarly, PSI was applied to the detection of steroids in wastewaters, detecting the hormones algestone acetophenide and levonorgestrel.^96^ He *et al* used nebulization DBD ionization to analyze acenaphthene in various water samples, including rainwater and water collected from local rivers.^97^ Acenaphthene is a polycyclic hydrocarbon commonly used in organic synthesis and as a fungistatic agent, and is a common contaminant encountered in water and soil. Using this method, the hydrocarbon could be detected down to sub‐ng/L levels with an analysis time of 5 minutes. Liu *et al* took a different approach to water analysis, using EESI for the detection of organophosphorus pesticides in environmental water samples. In this study, magnetic‐zirconia nanocomposite enrichment was conducted prior to analysis to improve the sensitivity and selectivity of the technique.^98^

Anatoxins are a class of cyanobacterial neurotoxins and are a concerning potential contaminant in drinking water reservoirs. In a study by Beach *et al*, a rapid DART‐HRMS method was developed for the analysis of cyanobacteria cultured in water, with triplicate analyses possible in <2 min.^99^ DART‐MS has also been employed in the detection of phthalic acid esters (PAEs), estrogenic endocrine disruptors, in water that have previously been detected in water supplies.^100^ Coupled with sorbent pre‐concentration and solvent‐assisted desorption, DART‐MS enabled the detection of PAEs at sub‐nanogram per liter levels. In recent years, environmental contamination by plastics has become a major concern to the health of both human beings and wildlife. Zhang *et al* recently used DART‐HRMS for the detection of microplastics in lakewater.^101^ After optimizing the technique for the analysis of 21 plastic samples, including synthetic fibers and microbeads from personal care products, the authors demonstrated a high‐throughput method that could be used to characterize plastics present in the environment and establish the type of plastics present in environmental samples.

AIMS has not only been used to detect environmental contaminants, but also to study the presence of such contaminants in living specimens. Wang *et al* used DESI‐MS and transmission electron microscopy to monitor the uptake and translocation of perfluoroalkyl substances (PFAS) by wetland plants.^102^ Eight common wetland plants were fed a nutrient solution spiked with perfluorooctanoic acid and perfluorooctanesulfonic acid, two common PFASs. The roots and stems of the plants were subject to analysis by DESI‐MS to study the distribution of the contaminants throughout the plant, providing invaluable insight into the interaction between vegetation and organic contaminants. Fipronil is a widely used insecticide with serious health implications in humans, thus its presence in the environment and in wildlife may require chemical monitoring. AP‐MALDI was recently applied to the imaging of slices of whole zebrafish that had previously been exposed to fipronil.^103^ In the comparison of untreated zebrafish and zebrafish exposed to the insecticide, significant differences were found in the phospholipid profile, suggesting that exposure to insecticides may result in changes in phospholipid metabolism.

The monitoring of contaminating particulate matter in indoor air has also been a subject of study in the application of AIMS. In a recent paper by Brown *et* al, EESI was used to measure volatile and semi‐volatile species in a simulated house environment, detecting a range of contaminants, including numerous siloxanes and phthalates.^104^ EESI has also been developed for the *in situ* detection of aerosol metal emissions, particularly toxic components produced during energy conversion and industrial processes.^105^ In this study, EESI was found to be capable of the rapid and high sensitivity detection of 34 different metal compounds, further demonstrating its potential for real‐time air monitoring.

### 3.6 Single‐cell analysis

Cells are the fundamental building blocks of all living organisms, containing a variety of analytes ranging from small metabolites to proteins. The ability to study the precise chemical composition of individual cells is essential in understanding cellular metabolism. In recent years, the use of mass spectrometry in single‐cell analysis has gained significant interest.^106^

Zhang *et al* used dispersed solid‐phase microextraction to extract phospholipids from two types of human leukemia cell prior to analysis by internal EESI‐MS.^107^ A series of protonated phospholipids were detected from the single cells, though with relatively low abundance due to the minute size. Following analysis, multivariate analysis was used to classify the different subpopulations of cells based on their chemical profiles. Infrared matrix‐assisted laser desorption electrospray ionization mass spectrometry (IR‐MALDESI‐MS) has also been applied to the analysis of lipids in single cells.^27^ HeLa cells were deposited on glass slides and subjected to direct analysis, resulting in the detection of 45 lipid species, primarily cholesterol and PCs. With no required sample pre‐treatment and an analysis time of approximately 1 min per cell, the study demonstrated the potential of MALDI techniques for rapid single‐cell lipidomics.

**FIGURE 4:**
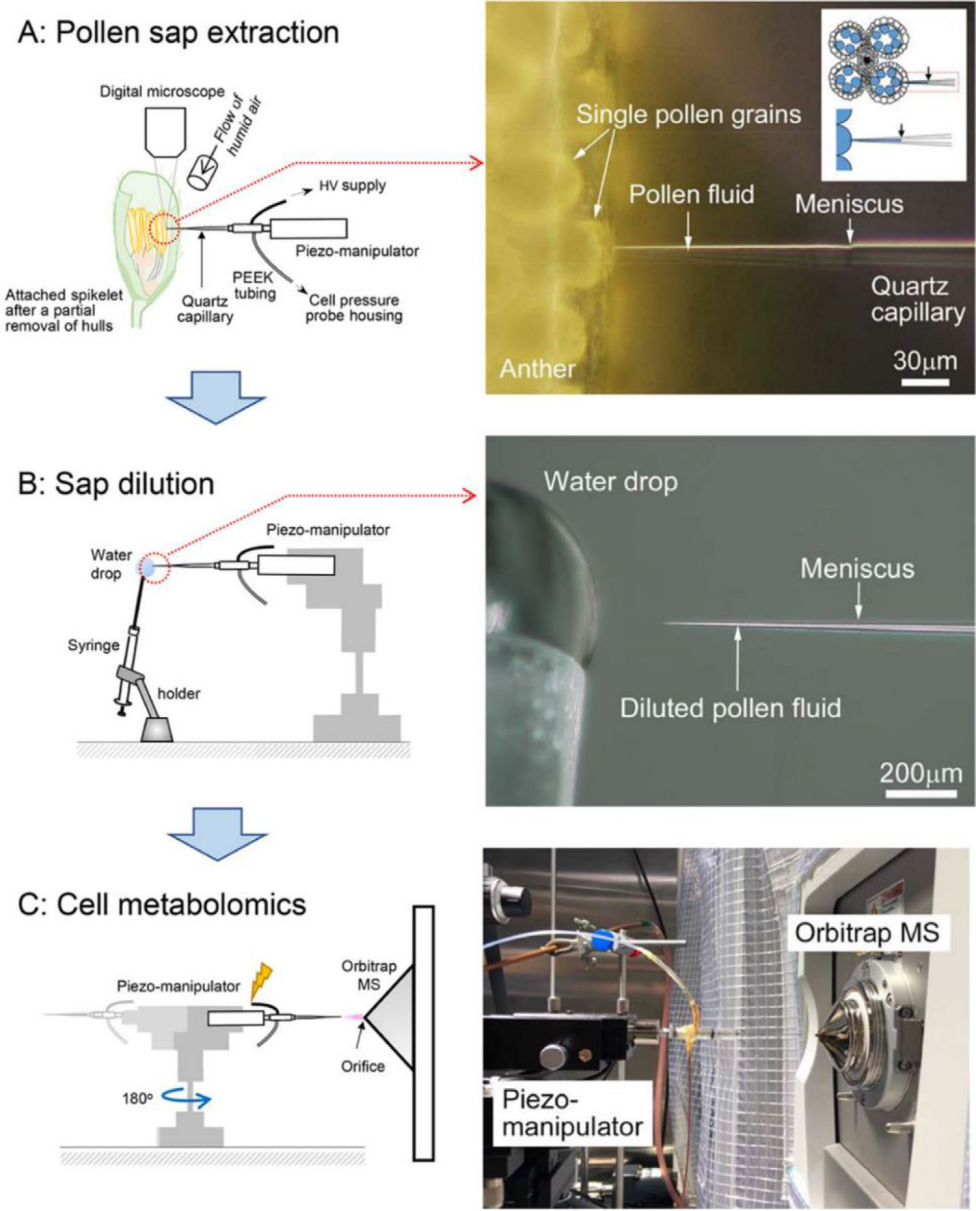
Workflow showing the on‐site analysis of cellular fluids from a single pollen grain. The tip is inserted into the pollen for the extraction of fluids from single grains (A). The probe tip is placed into a water droplet for dilution (B), after which the probe tip is positioned in front of the mass spectrometer inlet using a micro‐manipulator and a high voltage applied (C). Reproduced under Creative Commons CC‐BY license from Wada *et al* 2020^109^

Recently, PESI has been demonstrated as a promising technique for single‐cell analysis. Zheng *et al* modified the traditional PESI method using reduced graphene oxide with a copper probe, intended to induce the enrichment of trace components from the single cell.^108^ This resulted in an improvement in both ionization and transmission efficiency. The technique, dubbed functional probe ESI, was applied to the analysis of neurotransmitters in PC12 cells, which are derived from the rat adrenal medulla. Both neurotransmitters and smaller metabolites, including amino acids and lipids, were detected. PESI was also utilized for the on‐site analysis of single pollen grains, which can be both unicellular and multicellular. A recent study by Wada *et al* applied picolitre pressure‐probe electrospray ionization (picoPPESI), a single‐cell metabolomics technique, to the analysis of developing pollen grains in intact plants, particularly focusing on the detection of metabolites related to heat tolerance.^109^ A Piezo manipulator was inserted into the pollen grains to collect cellular fluid, which was subsequently directly analyzed by HRMS (Figure 4). Dozens of metabolites were detected, including plant growth hormones such as gibberellin A3 and trans‐zeatin, and heat‐dependent metabolites such as glycerol‐3‐phosphate. This technique was readily applicable to the analysis of live plants, enabling onsite and real‐time pollen metabolomics.

**FIGURE 5:**
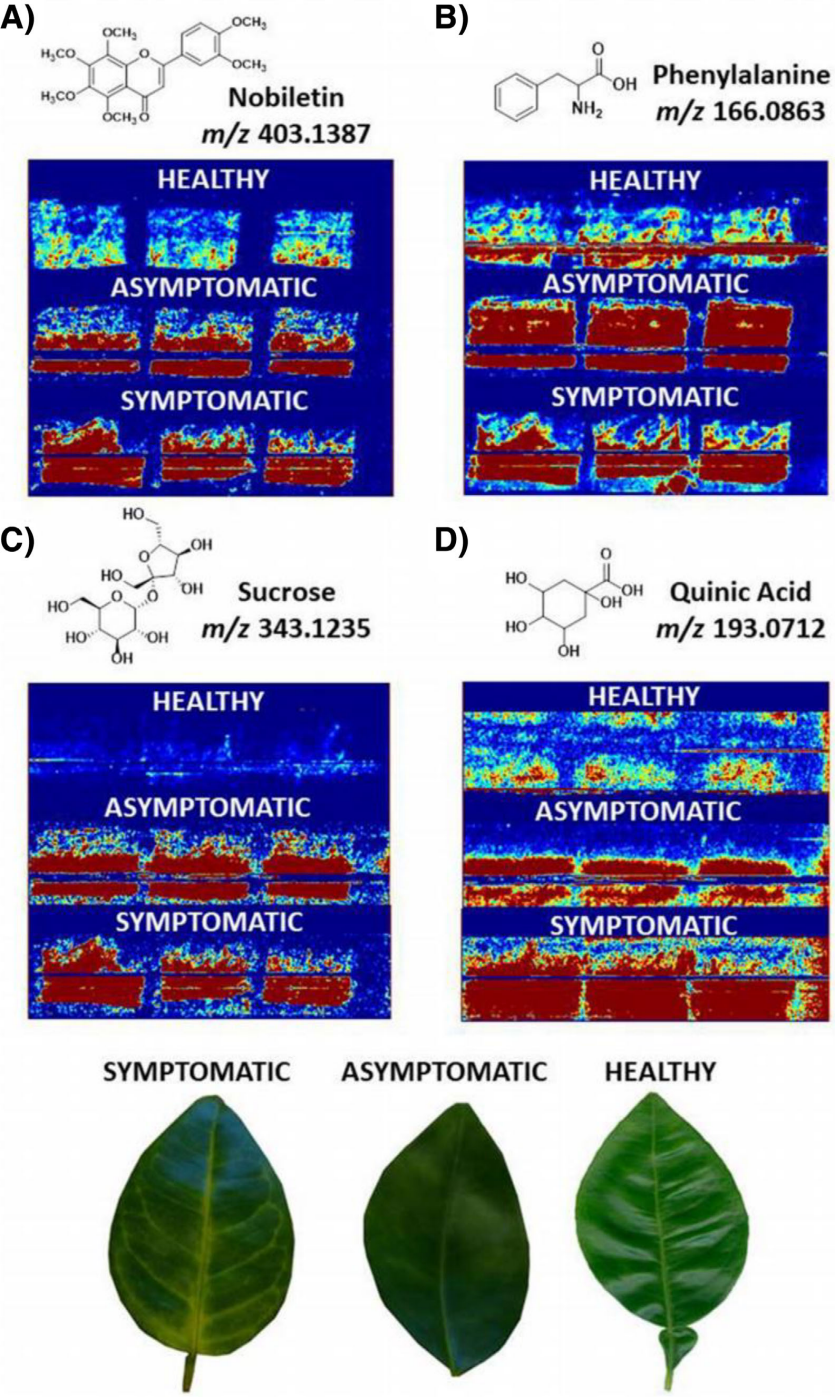
DESI imaging of Huanglongbing disease biomarkers in asymptomatic and symptomatic leaves in comparison to healthy leaves. Reproduced under Creative Commons CC‐BY license from de Moraes Pontes *et al* 2020^114^

### 3.7 Biological material

A primary benefit of AIMS techniques is the ability to analyze complex matrices without the need for prior sample preparation. This makes AIMS particularly suited to the analysis of complex biological materials, ranging from microbes to plant and animal tissues. Identification of bacteria typically relies upon time‐consuming and unreliable phenotypical techniques and, more recently, molecular methods. Recently, several studies have harnessed the high‐throughput nature of direct analysis mass spectrometry to analyze bacteria for the purpose of differentiating between different strains and developing rapid identification protocols.

Lellman *et al* described the use of AP‐MALDI for the analysis of genetically diverse bacterial strains, including *Staphylococcus aureus*, *Klebsiella pneumoniae*, and *Escherichia coli*, all of which have significant clinical relevance.^110^ Each species was found to produce a unique lipid profile in the *m/z* 400–1100 range, and multivariate analysis enabled species classification with an accuracy of 98.63%. A particularly interesting application of ambient ionization in bacteria analysis was performed by Havlikova *et al*, focusing on the detection of ESKAPE pathogens, which are the leading cause of hospital‐acquired infections.^111^ The study developed living 3D skin models to simulate wounds onto which ESKAPE pathogens were deposited, cultured, and subsequently analyzed by LESA‐MS for the detection and identification of proteins specific to different types of bacteria. This rapid approach was able to differentiate between closely related strains of bacteria, providing clinical staff with real‐time and dynamic information regarding bacteria type and behavior, potentially enabling the improved diagnosis and treatment of wound infections. Lee *et al* used SESI coupled with HRMS to study the influence of the antibiotic ampicillin on gut microbiota.^112^ Fecal samples from healthy volunteers were extracted and used to culture gut microbiota, some of which were subjected to ampicillin treatment. Headspace volatiles were directed from culture plates into the SESI ion source for direct and rapid ionization, with a particular focus on fatty acids. Numerous changes were observed after treatment with antibiotics, including the elevation of C_4_ and C_7_ fatty acids. SESI‐MS was shown to be a rapid and sensitive technique for studying changes in the gut microbiome. A particularly interesting approach was taken by Kocurek *et al* in the analysis of intact proteins from living yeast colonies. The thick chitin‐containing cell walls of yeast can make cell lysis prior to analysis difficult. In this study, electroporation was used to achieve the rapid release of intact proteins from yeast cells, followed by LESA‐MS analysis. The approach could be applied directly to colonies on agar plates, and could feasibly be coupled with other ambient ionization techniques.^113^

The application of ambient ionization mass spectrometry to living organisms is by no means restricted to small microbes, but is readily extended to the analysis of more complex biological tissues. Huanglongbing is a problematic disease known to affect different species of citrus, resulting in the formation of defective fruits, mottled leaves, and twig dieback. Due to the potential commercial implications of this, researchers have explored the use of mass spectrometry imaging to rapidly detect metabolites that may be indicative of the disease. DESI‐MSI was applied to the analysis of healthy and infected leaves, with minimal required sample preparation.^114^ The samples were also analyzed by LC–MS/MS for means of method comparison and to assist in compound identification. Numerous metabolites were found to increase in diseased samples, including quinic acid, sucrose, phenylalanine, and nobiletin (Figure 5). Furthermore, mass spectral imaging allowed for the study of metabolite distribution throughout the leaves, which could prove beneficial in understanding the extent and spread of infection.

AIMS technologies have been increasingly applied to animal tissues with minimal or no sample preparation, particularly tissues harvested from mice. The application of PESI‐MS to animal tissues has been frequently demonstrated by Zaitsu *et al*, who have developed a PESI‐MS technique for the high‐throughput direct metabolomic analysis of murine tissue samples.^115^ In the group's most recent study, PESI‐MS/MS was applied to mouse liver and brain samples, focusing on the detection of 72 metabolites related to energy metabolism. The mass analysis was coupled with a new data analytics platform, referred to as PiTMaP, which rapidly performs multivariate analysis such as PCA alongside other statistical analyses, and generates figures such as box‐and‐whisker plots for all metabolites. In all, the preparation‐free analysis time and post hoc data analysis takes approximately 1 min, introducing a powerful new platform for high‐throughput tissue metabolomics. Laser ablation techniques have also been applied to the analysis of murine tissues. LAAPPI and LAESI were recently utilized in the imaging of rodent brains.^116^ A new infrared laser focusing technique was coupled with ambient mass spectrometry and applied to the analysis of lipids and other metabolites in dissected mouse brain samples, achieving tissue imaging with sub‐100 μm spatial resolution. Conversely, Cordeiro *et al* used a DESI‐MS approach to the analysis of animal tissues, specifically targeting lipids in mammalian ovaries.^117^ Ovarian tissue from cows, sows, and mice at different physiological stages were studied, imaging the lipid distribution throughout the tissues. Interestingly, the study showed that the lipid distribution in different areas of the tissue was similar, independent of the species, suggesting lipid profiles are conserved across different species. Lin *et al* applied DESI and nanoDESI imaging combined with MALDI‐MSI and optical microscopy for the analysis of lipids and proteins in murine brain and kidney tissues and cancerous human tissues.^118^ With a spatial resolution of 5 μm and 100 μm for DESI/nanoDESI and MALDI, respectively, the multimodal tissue imaging approach enabled improved precision in the determination of tumor margins, along with the detection of several new potential tumor biomarkers. Finally, LESA‐MS has also been widely applied to the study of animal tissues, particularly for the detection of proteins. Cooper *et al* have applied LESA coupled with various forms of ion mobility spectrometry to the analysis of intact proteins in various biological tissues, including murine kidney, brain, and testes.^119^, ^120^, ^121^, ^122^

The use of ambient ionization mass spectrometry in animal tissue analysis has not been limited to omics studies, but has also proven useful in the analysis of pharmaceutical residues in biological tissues. A major human health concern is the presence of drug residues and other contaminants in animal tissues intended for human consumption, and direct analysis techniques are crucial for ensuring rapid and cost‐effective monitoring of animal products prior to distribution. CBS has been utilized for the detection and quantitation of drugs in bovine tissue, introducing a thin layer of biocompatible polyacrylonitrile onto the CBS device to improve the biocompatibility of the spray blade.^123^ Over 100 veterinary drugs in homogenized bovine tissue were detected and quantified using internal standards, achieving sample analysis in as short as 1 minute per sample and typically yielding RSDs of less than 25%. A different approach to this challenge was taken by Lu *et al*, who used DBD to detect drug residues in live fish.^124^ This study utilized a tungsten probe to penetrate the sample prior to DBD ionization, and specifically focused on the detection of pyrimethamine, a drug used for the treatment and prevention of the malaria parasite *Plasmodium falciparum*. The technique demonstrated good reproducibility for an ambient ionization source, with an RSD of 8.3%, and the minimally invasive sampling approach enabled the analysis of live animals.

### 3.8 Reaction monitoring

In organic synthesis, the optimization of chemical reactions is a major bottleneck in need of automated rapid analysis techniques. In recent years, the potential benefits of AIMS to ease this bottleneck have become apparent.

DESI‐MS has been particularly utilized in the real‐time study of chemical reactions. Recently, the Cooks group has developed DESI‐based high throughput systems for the screening of different reaction conditions in small droplets. Reaction mixtures with varying parameters were applied to sampling plates and analyzed by DESI, achieving the analysis of thousands of samples in a day. This high throughput system enabled varying conditions in nucleophilic aromatic substitution reactions,^125^ N‐alkylation, N‐acylation, and N‐sulfonylation reactions to be monitored,^126^ and more recently reductive amination reactions.^127^ The same group has also applied DESI‐MS to the study of enzymatic reactions. Enzymatic assays are essential in the field of drug discovery, but typically require the use of labeled compounds and plate readers to study the reactions. In a recent study, Morato *et al* applied a high throughput DESI‐MS system for the rapid analysis of label‐free enzymatic reactions, achieving an analysis time of 0.3 s per sample.^128^ PESI‐MS has also been explored as a potential solution to the bottleneck in chemical reaction monitoring, though to a lesser extent. In one recent study, PESI‐MS was applied to the study of heterogeneous photocatalytic reactions.^129^ The PESI probe was surface‐modified by a photocatalyst to increase wettability and ensure consistency throughout the entire monitoring process.

Pagliano *et al* used DART‐HRMS for the real‐time study of mechanisms of chemical vapor generation.^130^ In comparison with analysis by GC‐MS, DART‐MS was able to detect a number of species not detected by traditional techniques, demonstrating ambient techniques are not only capable of significantly faster analysis, but also identifying previously undetected analytes. However, the authors did experience some interference in analyte identification due to oxidation occurring in the DART source. Masuda and Kobayashi similarly employed DART‐MS, this time for the rapid monitoring of direct‐type hydroxymethylation and Mukaiyama‐type hydroxymethylation reactions, achieving quantification using an isotope‐labeled internal standard.^131^ Finally, Ashton *et al* applied DART‐MS to study thermally‐driven chemical reactions.^132^ The method combined a heated stage onto which a few milligrams of the material was placed and heated in a linear fashion, with DART monitoring changes throughout the process. As a result, small‐scale thermally driven reactions could be monitored in real time.

### 3.9 Miscellaneous applications

Although the majority of AIMS studies fall into a handful of categories, a broad range of fields of research have profited from the benefits of ambient ionization mass spectrometry. Polymers are an essential material in the modern world, being a vital component in packing material, containers, toys, and clothing. During the production of polyamides, a popular polymer more commonly known as nylon, various undesired by‐products are formed due to thermal decomposition. Fourier‐transform infrared spectrometry (FTIR) and pyrolysis GC‐MS are the gold standard techniques for polymer analysis, however, both techniques require sample preparation. DART‐HRMS has been demonstrated to be a rapid and reliable tool for the differentiation and identification of polyamide polymers. In a recent study, ten types of polyamide polymer were introduced into the DART ion source for just 5 seconds, revealing the distinct mass spectra of the different sample types, with cyclic monomers and dimers dominating the mass spectra.^133^ In a similar study, Zughaibi and Steiner used DART‐TOF‐MS to differentiate between seven types of nylon, though the study only analyzed pure nylon standards as opposed to real‐world materials.^134^ Furthermore, Cody *et al* applied thermal desorption and pyrolysis DART to the analysis of industrial polymers.^135^ Mass spectra acquired from polymers exhibited a series of peaks that differ by the monomer mass, and Kendrick mass defect analysis was applied for the identification of characteristic peaks. Collectively, these studies have demonstrated the potential of DART‐MS for rapid polymer analysis.

The field of petroleomics involves the analysis and identification of chemical components in petroleum and its products. Due to the broad range of compounds present in petroleum, extensive sample preparation is typically required when using traditional MS techniques to avoid instrument contamination. DBD‐MS was recently applied to the rapid analysis of automotive lubricant, a type of lubricant made from crude oil.^136^ A total of 35 commercially‐available oil samples were acquired, consisting of a combination of synthetic, semi‐synthetic, and mineral oils. Small aliquots of unprocessed oil were exposed to the DBD plasma for mass spectral analysis, after which multivariate analysis was employed to differentiate between the sample types. Despite the highly complex mass spectral profiles acquired, multivariate analysis enabled the detection of characteristic mass patterns for different brands and viscosities, which could prove beneficial in both authentication and quality control of automotive lubricant oils.

Tega *et al* described the use of venturi (V)‐EASI for the analysis of fuels.^137^ In recent years, the adulteration of gasoline and biofuels with methanol has become common practice, resulting in the need for rapid analytical protocols for sample authentication. Gasoline, ethanol fuel, and biodiesel were acquired from service stations, and the detection and quantification of methanol were rapidly achieved by V‐EASI, with an analysis time of 30 s per sample. Although V‐EASI offers a rapid solution to fuel analysis, on this occasion authors carried out derivatization prior to analysis to improve ionization of methanol.

Gasoline performance can be modified by the addition of certain additives, though stringent quality control procedures are required to monitor the formulations. ASAP‐MS has been applied in the field of proteomics to study such additives. ASAP and ASAP coupled with high‐performance thin‐layer chromatography (TLC) were used for the detection of polymeric additives in gasoline.^138^ Samples were analyzed either directly or following chromatographic separation, the latter being first subjected to TLC to separate gasoline components, after which a moistened glass capillary was scratched across the surface of the TLC plate. The fast and user‐friendly method enabled rapid detection of additives, though the authors noted that the technique was suitable for neither high‐throughput nor quantification.

Consumer goods, such as clothing and cosmetics, are often the subject of stringent regulations regarding the presence and levels of potentially hazardous substances. Parabens are a group of parahydroxybenzoates commonly added to cosmetics and pharmaceutical drugs. Although parabens are added to goods due to their antimicrobial properties, they are also weak endocrine disrupters, thus their presence in certain products must be regulated. Bartella *et al* used PSI‐MS for the rapid analysis of parabens in consumer products.^139^ Various commercially available products were analyzed, including makeup cream, anti‐aging cream, toothpaste, and cough syrup. The study described a rapid and reliable technique for the detection of parabens in a range of matrices, with RSD values lower than 15%. In this case, the parabens detected were similar to those present on product labels, indicating none of the products exceeded limits implemented by regulatory agencies.

The manufacture of textiles frequently involves the use of potentially harmful chemicals, and concerns have been raised over the extent to which such substances remain in the materials after production.^140^ Amongst the substances of concern are perfluorinated carboxylic acids (PFCAs), a class of chemicals widely applied to textiles for the repellence of dirt and oil. Wang *et al* developed a DBD‐MS/MS method for the rapid analysis of PFCAs in textiles, applying the technique to the direct analysis of 15 real‐world textile samples, including wool, cotton, nylon, and polyester.^140^ Only one of the real‐world samples was found to contain a PFCA, but quantification of the analyte was in line with analysis by HPLC‐MS/MS, highlighting the reliability of DBD for the quantification of textile contaminants.

**FIGURE 6:**
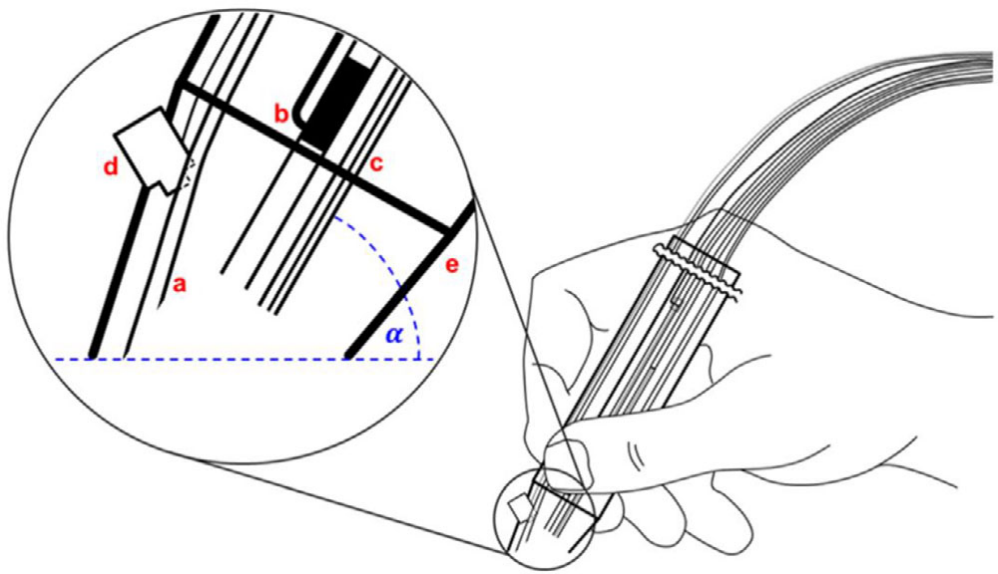
Schematic diagram of the 3‐in‐1 hand‐held device, showing the droplet collection tube (A), the low‐temperature plasma emitter (B), the DESI and EASI emitter (C), the camera (D), and the probe housing (E). Reproduced with permission from The American Chemical Society from Meisenbichler *et al* 2020^141^

Finally, some groups have taken steps to improve the usability of AIMS techniques, with the aim of developing user‐friendly and portable technology that can be utilized in a range of scenarios. Meisenbichler *et al* developed a hand‐held, pen‐like ambient ionization device combining LTP, DESI, and EASI ion sources suitable for the direct surface desorption/ionization of analytes with a range of chemistries (Figure 6).^141^ The device is simply placed against the surface of the sample with the desired ionization mode activated, and produced ions are transported to the mass spectrometer via a 60 cm transfer line. A range of samples was tested with the device, including pharmaceuticals, food products, and individual chemical standards. In another recent study, Jager *et al* described a USB‐powered coated blade spray ion source for on‐site analysis.^142^ The small device costed only $10 to manufacture and could be coupled with smartphones and tablets for the generation of the high voltage, or low‐to cost high‐voltage generators. The device was coupled with both a benchtop triple quadrupole mass spectrometer and a transportable single quadrupole instrument and assessed with various sample types. Studies such as these have highlighted the potential to create versatile and easy‐to‐use AIMS devices that can be readily utilized in the laboratory or on‐site, which has great potential in various fields of work.

## 4 SUMMARY AND OUTLOOK

The introduction of ambient ionization has revolutionized analytical chemistry, allowing sample analysis to be conducted in a faster, simpler, and more efficient manner. Each year interest in AIMS grows, demonstrated by the rapid increase in new techniques and the ongoing developments to improve the performance of existing techniques. This review demonstrates that the use of AIMS techniques has been observed across a broad range of fields, from biomedical applications for improved disease diagnostics, to forensic science to aid the criminal justice system, and rapid food analysis to ensure the health and safety of consumers. Despite the broad potential applications of AIMS, currently ambient ionization techniques are largely confined to the realms of research and development, principally due to some of the inherent challenges associated with its adoption for widespread and certified use. Before many of these techniques can become commonplace in clinical and industrial labs, scientists must first overcome a number of weaknesses, particularly relating to reproducibility, sensitivity, quantitative capabilities, and ion suppression.

As techniques in this field continue to be developed and refined, new and exciting applications will inevitably emerge, which will ensure AIMS becomes widely employed in academic research, industry, and society as a whole.

## Data Availability

Data sharing not applicable to this article as no datasets were generated or analyzed during the current study.
